# Construct and Criterion Validity of the Postmenopause Sexuality Questionnaire – PMSQ

**DOI:** 10.1055/s-0040-1701461

**Published:** 2020-01

**Authors:** Maria José Ferreira Lima, Marília Duarte Valim, Sebastião Freitas de Medeiros

**Affiliations:** 1 Department of Gynecology and Obstetrics, Júlio Muller University Hospital, Cuiabá, MT, Brazil; 2 School of Nursing, Universidade Federal de Mato Grosso, Cuiabá, MT, Brazil; 3 Department of Gynecology and Obstetrics, Faculdade de Ciências Médicas, Universidade Federal de Mato Grosso, Cuiabá, MT, Brazil

**Keywords:** menopause, sexuality, physiological sexual dysfunction, psychometric, sensitivity, specificity, menopausa, sexualidade, disfunção sexual fisiológica, psicometria, sensibilidade, especificidade

## Abstract

**Objective** To assess the construct and criterion validity of the Postmenopause Sexuality Questionnaire (PMSQ).

**Methods** The present methodological questionnaire validation study included postmenopausal women. The construct validity was tested by factor analysis and the criterion validity was performed using the correlation between the PMSQ and the Female Sexual Function Index (FSFI). The ROC curve was used to verify sensitivity, specificity and to determine the cutoff point of the PMSQ.

**Results** A total of 181 women with a mean age of 56.4 ± 5.7 years old were evaluated. The exploratory factor analysis showed that the PMSQ presented Kaiser test = 0.88 and χ^2^ = 3293.7 (*p* < 0.001), commonalities ≥ 0.5, and extraction of 9 factors with eigenvalue ≥ 1; explaining 66.3% of the total variance. The PMSQ presented factor loadings between 0.4 and 0.8. A strong correlation between the 2 questionnaires (r = 0.79; *p* = 0.000) was shown. The cutoff point of the PMSQ was ≤ 55.5, assuming 87.9% sensitivity and 78.9% specificity (*p* < 0.001).

**Conclusion** Since the PMSQ showed a strong correlation with the FSFI questionnaire, it presented good psychometric properties to assess the sexuality in postmenopausal women. Based on these results, the PMSQ could be widely tested as a specific instrument to examine the sexual function in postmenopausal women. Future studies, designed to examine the PMSQ instrument in different populations, are needed.

## Resumo

**Objetivos** Validar o construto e o critério do Questionário para Avaliação da Sexualidade Feminina após a Menopausa (QSFM).

**Métodos** Estudo metodológico de validação de questionário incluiu mulheres na pós-menopausa. A validade de construto foi testada por meio da análise fatorial e a validade de critério foi realizada por meio da correlação entre o QSFM e o Índice de Função Sexual Feminina (FSFI). A Curva ROC foi utilizada para verificar sensibilidade, especificidade e determinar o ponto de corte do QSFM.

**Resultados** Foram avaliadas 181 mulheres, com idade média de 56,4 ± 5,7 anos. A análise fatorial exploratória mostrou que o QSFM apresentou teste de Kaiser = 0,88 e χ^2^ = 3293,7 (*p* < 0,001), comunalidades ≥ 0,5 com extração de nove fatores com autovalor ≥ 1; explicando 66,3% da variância total. O QSFM apresentou cargas fatoriais entre 0,4 e 0,8. Uma forte correlação entre os dois questionários (r = 0,79; *p* = 0,000) foi demonstrada. O ponto de corte do QSFM foi ≤ 55,5, assumindo sensibilidade de 87,9% e especificidade de 78,9% (*p* < 0,001).

**Conclusão** Como o QSFM demonstrou uma forte concordância com o questionário FSFI, ele apresentou boas propriedades psicométricas para avaliar a sexualidade em mulheres na pós-menopausa. Com base nesses resultados, o QSFM pode ser amplamente utilizado como um instrumento específico para examinar a função sexual em mulheres na pós-menopausa. Estudos futuros são necessários para examinar o instrumento QSFM em diferentes populações.

## Introduction

The climacteric period is a biological phase of life and not a pathological process.^1^ Menopause is defined as the last menstrual period, recognized 12 months after its occurrence.^2^ The mean age of menopause ranges from 48 to 52 years globally.^3^ In this period, hormonal variations and progressive estrogen deficiency may sometimes result in debilitating short, medium and long term conditions.^4^ Decreased pelvic support, genital atrophy, and decreased lubrication may result in dyspareunia and finally in sexual dysfunction.^5^ Female sexual desire is not spontaneous and the sexual response includes intimacy and emotional satisfaction as goals.^6^ Any disruptions in the response cycle, such as dyspareunia or difficulties with arousal, reduce motivation and the search for intimacy with the partner. Sexual dysfunction can affect women of various ages, colors, social status and ethnicities. It is characterized by psychophysiological changes in the sexual response, including sexual desire, arousal, orgasm, and even pain.^7^ Hormonal changes in the menopause, particularly hypoestrogenism and decreased testosterone levels, associated with biological, cultural and social processes may have a direct impact on sexuality, well-being, and quality of life.^8^ ^9^ ^10^

The prevalence of sexual dysfunction after menopause has shown to increase from 12.1% to 48.0%.^11^ Worldwide, the prevalence of sexual dysfunction in postmenopausal women using questionnaires that do not include a specific menopause domain has been reported to vary between 61% and 86%.^12^ ^13^ In sexually active postmenopausal Brazilian women, it seems that 70% suffer from sexual dysfunction, especially those > 50 years old.^14^ ^15^ Among the studies on sexual dysfunction in older women, the most commonly used instrument is the FSFI and its short form FSFI–6, but other instruments have also been used.^16^ ^17^ ^18^ ^19^ Even though these instruments measure sexual dysfunction in menopausal, perimenopausal and postmenopausal women, they do not have a menopause specific domain, and they are not quite suitable for measuring sexual dysfunction in postmenopausal women. They do not have questions linking sexual dysfunction to the menopause condition.

The PMSQ tested in the current study was previously and partially validated in Portuguese to measure the different domains of sexual function in Brazilian postmenopausal women.^20^ Therefore, the primary objective of the present study was to assess the construct and criterion validities of this questionnaire and to determine its cutoff level to identify postmenopausal women with or without sexual dysfunction.

## Methods

This methodological study enrolled postmenopausal women who were selected using accessibility sampling at the General Gynecology and Climacteric Outpatient Clinics of a teaching and research hospital, between November 2017 and June 2018. According to the current recommendations, the sample size criterion for factor analysis was 5 subjects per item.^21^ ^22^ ^23^ ^24^ ^25^ ^26^ ^27^ ^28^ A total of 181 postmenopausal women with stable and regular sexual activity, regardless of marital status or sexual orientation, were examined. Natural menopause was defined as 12 consecutive months of absence of menstruation. Women with hysterectomy before menopause were included if age ≥ 48 years old and follicle stimulating hormone (FSH) ≥ 25 mIU/mL.^3^ Other hormones such as estradiol, total testosterone, free thyroxin, and thyroid stimulating hormone were also measured. Women with an earlier diagnose of menopause, already using estrogen-progestin hormone therapy, were also included. Women with severe hypertension, decompensated diabetes, severe heart disease, musculoskeletal diseases with movement disabilities, current or past cancer diagnosis, bilateral oophorectomy, vulvodynia or using medications that could interfere with the libido were excluded.

Data were collected during a single interview, after signing the free and informed consent form. Sociodemographic characteristics, body weight, and height were obtained with the woman standing barefoot. The body mass index (BMI) (weight / height^2^) was calculated following the Brazilian guidelines.^22^ ^23^ The waist circumference was verified using an inelastic tape, positioned at the smallest circumference between the final costal arch and the iliac crest. The PMSQ and the FSFI questionnaires were both applied in this sequence and face to face. Despite the referred formal educational level, most of the patients had very little schooling and little ability to read and answer the questionnaires without help. So, a single researcher, the main author, carefully read the questions and the participant pointed out the item that corresponded to the answer she had chosen. Approval of the project was obtained from the local Ethics and Research Committee.

The PMSQ initially contained 43 items distributed into nine domains, namely: self-image (5), sexual quality of life (6), sexual intimacy (6), desire (7), arousal (5), orgasm (4), dyspareunia/vaginism (2), satisfaction (5) and influence of menopause (3). The questions were answered on an ordinal Likert scale (0–5). The scores (0–100) were standardized by the formula (X / 215) × 100, where: X is the answer for each item and 215 is the maximum possible gross score (5 × 43 = 215); 0 indicates the worst sexual function and 100 indicates the best sexual function. The items were designed based on sexual domains validated in other questionnaires, and all of them were evaluated by specialists in sexology and submitted to the test-retest method.^20^ For the publication in English, the PMSQ instrument was translated from Portuguese into English as follows: a native English speaker and a native Brazilian Portuguese speaker translated the questionnaire independently. Finally, a bilingual author confronted the two versions, keeping the most appropriate terms. As the instrument was applied to women of native Portuguese language, the English version of the instrument was not yet validated in any English speaking population.

The FSFI, a gold standard questionnaire designed to evaluate female sexual function, was previously validated in Brazilian Portuguese.^24^ This instrument contains 19 items in six domains: sexual desire (2), sexual arousal (4), vaginal lubrication (4), orgasm (3), sexual satisfaction (3) and pain (3). The items are answered on an ordinal Likert scale (0–5), with increasing scores according to the presence of the function questioned, with total scores varying from 2 to 36. Based on validation studies, a cutoff point of 26.5 was proposed.^25^ However, its cutoff point to discriminate menopause women with or without sexual dysfunction was established as 23.^26^ Because the FSFI has been used in populations of all ages, including menopausal women, and already presented a cutoff considering the age, it was chosen for validating the PMSQ questionnaire.

Descriptive analyses of data included the variable ages, family income in minimum wages, education, self-declared color, occupation, number of previous pregnancies, menarche age, sexarche age, menopause age, BMI, waist-hip ratio, clinical comorbidities and use of menopause hormone therapy. The data distribution was verified using the Shapiro-Wilk test. The Cronbach α was used to verify the internal consistency of both PMSQ and FSFI questionnaires. The Pearson coefficient correlation was used to verify the possible correlation between PMSQ and FSFI questionnaires. Exploratory Factor Analyses were used to examine the construct validity of the PMSQ. The Kaiser-Mayer-Olkin test measured the fitness of the sample and the Bartlett sphericity test verified whether the data were adequate for the analysis; Varimax orthogonal rotation principal component analysis was used, and any factor loading > 0.40 was retained for interpretation of the instrument structure.^27^ ^28^

The criterion validity was performed using the Pearson correlation coefficient (r), between the PMSQ and FSFI as gold standard.^29^ The cutoff point of the PMSQ questionnaire was established using the ROC curve with a 95% confidence interval (CI). The scores ≤ 23, suitable for women > 50 years old, were used as cutoff points of the FSFI.^26^ ^30^ Proportions between two variables estimated by the FSFI and PMSQ were compared using the chi-squared test (χ^2^). The data and this exploratory factorial analysis were performed using the SPSS Statistics for Windows, version 17 (IBM Corp., Armonk, NY, USA). The ROC curve was calculated using the Medcalc Statistical Software version 18.9.1 (MedCalc Software, Ostend, Belgium). Any p-value < 0.05 was considered statistically significant in a two-tailed test.

## Results

Most participants were married (85.1%) and more than half (60.8%) self-declared as catholic. As for self-declared color, 58.0% (105) were mixed, 28.2% (51) white, and 13.8% (25) black. More than half (53.0%) had a maximum of 8 years of schooling. Almost two-thirds of them (61.3%) gained a family income of 2 minimum wages, that is, about £400 per month. The BMI was 29.1 ± 5.0 kg/m^2^. A total of 96 (53.0%) subjects reported regular physical activity, with a mean of 3.3 ± 1.2 times a week. Smoking was reported by 8.8% (16/181) and 21.0% (38/181) reported drinking socially; ∼ 1 beer (500ml) per week. The mean ages of menarche and sexarche were 13.3 ± 1.7 years old and 19.3 ± 3.9 years old, respectively. Women with previous hysterectomy (27.1%) reported surgery at 41.8 ± 7.0 years old. The age of natural menopause in 132 participants (72.9%) was 48.4 ± 5.2 years.

The descriptive analysis of the PMSQ questionnaire with 43 items yielded a mean score of 54.9 ± 15.1 and total α coefficient of 0.93; in the domains self-image and dyspareunia, the α coefficients were 0.44 and 0.33, respectively. The correlation matrix of the PMSQ with 43 items showed significant correlations between all items, but the item 34 (I can put my finger in my vagina without feeling pain) presented poor, but still significant correlations (r < 0.30), and a sampling adequacy measure of 0.42 in the anti-image matrix; therefore, this item was removed from the exploratory factor analyses. The remaining 42 items presented Kaiser's test = 0.88, χ^2^ = 4006, *p* < 0.001, indicating that the sample and the correlation matrix were adequate to carry out the exploratory analyses. Almost all items presented commonalities ≥ 0.5 and 10 factors were extracted with an eigenvalue ≥ 1, explaining 66.06% of the total variance.

Afterwards, rotated analyses of the matrix enabled the exclusion of 6 items: item 29 (it is very difficult for me to get aroused, loadings ≤ 0.4); item 22 [(I get aroused just by thinking about having sex) loading in the same factor as item 25 (I get excited just thinking about sex)]; item 3 (I am sexually desirable), item 8 (my partner's sexual performance satisfies me), item 11 (I feel frustrated about my sexual life) and item 17 (I like my partner to caress my genitals [vagina, clitoris]) because they loaded in more than one factor. The item 19 (I like to have sex/make love), despite presenting cross loadings in three factors, was maintained because it is clinically important to assess the postmenopausal sexuality.

After deleting the previously mentioned items, a new exploratory analysis of the PMSQ with 36 items was performed. The analysis of this 36-item version showed Kaiser test = 0.88 and χ^2^ = 3293 (*p* < 0.001) and commonalities ≥0.5 (Table 1). Nine factors were extracted with eigenvalue ≥ 1, explaining 66.3% of the total variance. After the rotation, item 23 (I feel pleasure during sexual intercourse) and 32 (I get easily aroused when I am touched), despite having crossed loads, they were maintained because they are clinically important in evaluating postmenopausal sexuality.

**Table 1:** Values of factor load and commonalities of the 36-item PMSQ^a^

	Items	Factors									
		1	2	3	4	5	6	7	8	9	h^2^
Orgasm	I only get an orgasm with great effort	0.84	0.11	0.12	0.10	0.04	0.25	0.09	0.12	0.02	0.82
	It is difficult for me to get an orgasm	0.78	0.20	0.20	0.14	0.11	0.23	0.14	0.12	−0.01	0.80
	I get an orgasm easily	0.74	−0.03	0.30	0.15	−0.05	0.04	0.23	0.16	0.09	0.73
	It is impossible for me to have an orgasm	0.65	0.13	0.14	0.27	0.13	0.21	0.07	0.08	0.12	0.62
Sexual intimacy	I hug and caress my partner's body during the intercourse	0.25	0.72	0.14	0.21	−0.02	−0.06	0.11	0.02	0.09	0.67
	I like to caress the penis of my partner	0.09	0.72	−0.13	0.31	0.22	0.09	0.02	0.00	−0.03	0.69
	I get emotionally involved with my partner during the sexual intercourse	0.15	0.69	0.21	0.14	0.19	0.09	0.03	0.17	0.06	0.64
	I like to be caressed by my partner	−0.01	0.69	0.26	−0.01	0.05	−0.02	0.19	−0.06	0.07	0.59
	I am concerned with my sexual life	−0.14	0.48	−0.12	−0.15	0.35	0.06	0.03	−0.35	0.06	0.54
	I am satisfied with my sentimental life	0.07	0.42	0.25	−0.10	0.03	0.05	−0.04	0.26	0.40	0.48
Satisfaction	The feeling of sex is good	0.16	0.12	0.72	0.25	0.22	0.11	0.19	0.04	0.06	0.72
	Sex makes me feel accomplished	0.32	0.21	0.67	0.26	0.07	0.13	0.02	0.12	0.13	0.71
	I feel satisfied with sex	0.24	0.20	0.63	0.32	0.18	0.19	0.17	0.09	0.10	0.71
	Considering the frequency of the relations with my current partner, I am	0.23	0.38	0.53	−0.13	0.03	0.16	−0.03	0.28	0.13	0.61
	I get easily aroused when I'm touched	0.17	0.10	0.46	0.43	0.19	0.22	0.17	−0.08	0.21	0.59
	I feel uncomfortable during the sexual intercourse	0.25	−0.07	0.44	0.08	−0.21	0.08	0.31	0.10	−0.23	0.48
Arousal	I get wet during the intercourse	0.25	0.01	0.14	0.69	0.01	0.07	0.01	0.06	−0.06	0.57
	I want to have sex	0.29	0.14	0.14	0.64	0.20	0.05	0.24	0.22	0.09	0.69
	I like to have sex/make love	0.14	0.39	0.18	0.61	0.22	0.13	0.30	0.05	0.04	0.74
	I feel like having sex when I am caressed	−0.07	0.24	0.33	0.51	0.17	0.16	0.39	−0.01	0.08	0.63
	I feel pleasure during sexual intercourse	0.43	0.18	0.34	0.47	−0.03	0.26	0.21	0.08	0.07	0.67
Desire	I get excited just by thinking about sex	0.08	0.03	0.10	0.27	0.77	0.19	−0.03	0.09	0.11	0.74
	I think, fantasize, dream of having sex/making love	−0.01	0.26	0.10	0.25	0.74	0.07	−0.05	0.04	0.10	0.70
	I really want to get sexually excited	0.11	0.15	0.21	0.01	0.66	−0.10	0.18	−0.10	−0.01	0.57
	I have less sexual intercourse than I would like to	0.01	0.01	−0.16	−0.23	0.51	−0.09	0.31	−0.27	−0.03	0.52
Menopause	The fact that I no longer menstruate increased the frequency of my sexual intercourse	0.18	−0.06	0.13	0.06	0.04	0.80	0.05	0.09	−0.08	0.71
	As a result of the menopause I feel less willing to have sex	0.24	0.16	0.09	0.08	0.03	0.77	0.18	0.08	−0.09	0.74
	How has your desire to have sex after menopause	0.23	0.02	0.24	0.22	0.02	0.65	0.23	0.20	0.01	0.68
Importance of sexual life	I want to have sex	0.12	0.12	0.09	0.27	0.24	0.21	0.65	0.03	0.13	0.65
	I am not interested in sex	0.30	0.16	0.25	0.21	0.14	0.15	0.63	0.15	0.05	0.68
	I feel sexually cold	0.37	0.12	0.18	0.11	−0.08	0.29	0.59	0.26	0.01	0.69
Quality of sexual life	I am unhappy with my sexual activity	0.11	0.11	0.17	0.04	0.04	0.03	0.27	0.77	−0.02	0.71
	I want to improve my sensuality	0.10	−0.15	−0.16	0.06	−0.14	0.21	−0.09	0.66	0.09	0.57
	I feel dissatisfied with my sexual activity	0.24	0.19	0.35	0.13	0.02	0.13	0.12	0.63	0.00	0.65
Self-image	I am still a sensual, charming woman	−0.01	0.15	0.08	−0.11	0.04	−0.04	0.23	−0.08	0.83	0.80
	I feel well with my body image	0.15	0.00	0.05	0.24	0.11	−0.12	−0.09	0.11	0.77	0.72

a Extraction method: principal component analysis; Rotation method: Varimax with Kaiser normalization.

h^2^ communality.

The items had factor loadings between 0.4 and 0.8, and total α of 0.92; among the domains, the α coefficients ranged between 0.63 and 0.87 (Table 2).

**Table 2:** Alpha coefficients of the total instrument and its specific domains (PMSQ)

Domain	Number of questions	Questions	Score	Cronbach α**
**Self-image**	2	1–2	0–5	0.63
**Quality of sexual life**	3	3–5	0–5	0.65
**Sexual intimacy**	6	6–11	0–5	0.75
**Desire**	4	12–15	0–5	0.68
**Importance of sexual life**	3	16–18	0–5	0.77
**Arousal**	5	19–23	0–5	0.82
**Orgasm**	4	24–27	0–5	0.87
**Satisfaction**	6	28–33	0–5	0.80
**Influence of menopause**	3	34–36	0–5	0.79
**Total**	36	–	–	0.92

** Cronbach α, measure of the internal consistency for each domain and the total questionnaire.

**Fig. 1:**
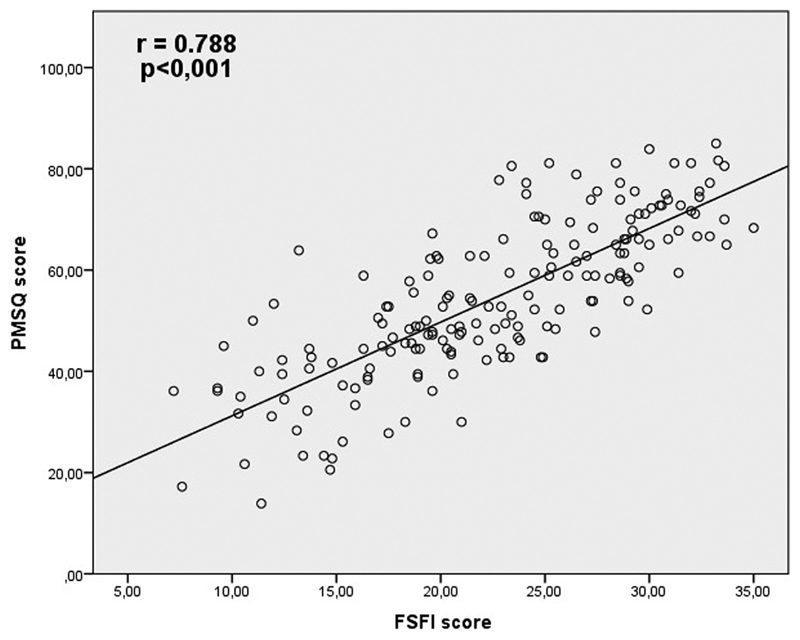
Correlation between FSFI and PMSQ scores.

The PMSQ, with 36 items, yielded a total mean score of 54.5 ± 15.4, whereas the specific menopause influence domain showed the lower mean (37 ± 24.5). The self-image domain presented the highest mean (65.9 ± 24.6). The average score obtained with the FSFI was 22.6 ± 6.5. The lowest mean was found in the desire domain (3.1 ± 1.2), and the highest one was found in the pain domain (4.2 ± 1.7). The FSFI presented a total α coefficient of 0.93 and, among the domains, the Cronbach α varied between 0.76 and 0.94. As shown in Fig. 1, the Pearson coefficient correlation between the two questionnaires was r = 0.788 (*p* < 0.001).

The association between the PMSQ and FSFI scores in the orgasm domain was strong (r = 0.70; *p* < 0.001). The influence of menopause showed moderate (r = 0.40–0.59; *p* < 0.001) correlations with all FSFI domains, except with the pain domain. The self-image domain, which was not evaluated in the FSFI, presented weak (r = 0.15–0.19) but significant correlations with all other domains of this questionnaire (*p* < 0.05 for all comparisons).

**Fig. 2:**
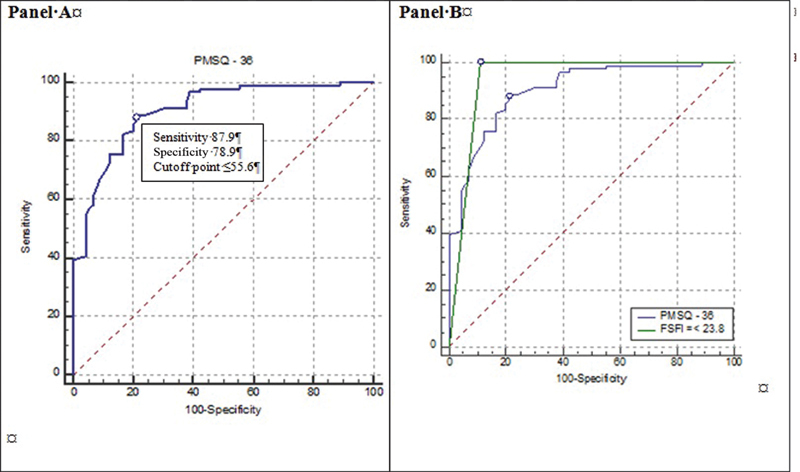
Panel A - ROC curve with cutoff point of PMSQ questionnaire. Panel B - Comparison between total areas of FSFI and PMSQ questionnaire.

The total score of ≤ 23 obtained in the FSFI was used as the classification variable. In the ROC curve analysis, an area under the curve of 0.90 (95% CI: 0.85–0.94) and cutoff point of ≤ 55.6 was observed in the PMSQ, and sensitivity of 87.9% and specificity of 78.9% (*p* < 0.001) was detected using this instrument (Fig. 2, panel A). Using the cutoff point of ≤ 23, the FSFI identified 91/181 (50.3%) women with sexual dysfunction. When the PMSQ questionnaire was used, 99/181 (54.7%) women reported sexual dysfunction. Therefore, regarding the ability to identify sexual dysfunction, no difference was found between the two questionnaires (4.42%; 95% CI: 5.82–14.53; χ^2^ = 0.71; *p* = 0.400). The areas of the ROC curves of the FSFI and PMSQ questionnaires were similar (difference of 4%; 95% CI: - 0.003–0.08; *p* = 0.07) (Fig. 2, panel B).

The comparison between proportions of menopausal women with sexual dysfunction and menopausal women without sexual dysfunction, either in the total or in a particular domain score in the PMSQ is shown in Table 3. It is worth noticing that because the primary objective was to validate this questionnaire, the analysis was not stratified by any patient characteristic.

**Table 3:** Comparison of the scores total and by domain of the PMSQ between women with and without sexual dysfunction

Domains	Without SD (82)	With SD (99)	T***	
	(sd)	(sd)		*p-value*
Orgasm	69.21 (20.99)	30.15 (18.74)	13.08	0.000
Menopause	51.63 (23.02)	24.85 (18.29)	8.54	0.000
Sexual intimacy	73.58 (15.63)	51.38 (18.93)	8.64	0.000
Quality sexual life	53.01 (20,84)	34.41 (19.28)	6.18	0.000
Self-image	72.68 (22.72)	60.20 (24.83)	3.53	0.001
Desire	53.66 (21.84)	38.99 (17.50)	4.91	0.000
Satisfaction	79.96 (8.60)	53.23 (17.86)	13.16	0.000
Arousal	71.95 (16.64)	40.69 (17.77)	12.20	0.000
Importance sexual life	77.97 (17.43)	45.86 (22.03)	10.94	0.000
Total	68.49 (7.45)	42.87 (9.36)	20.50	0.000

Abbreviations: SD, sexual dysfunction; sd, standard deviation.

*** Student's *t*-test.

## Discussion

The demographic profile of the study participants is similar to the profile already performed in other studies in Brazil and other countries.^12^ ^31^ ^32^ More than half (62.4%) of the participants were overweight or obese. About half of them exercised regularly and had concluded their fourth grade education, and more than half (61.3%) had a monthly family income of £400. The vast majority (85.0%) were married. Natural menopause occurred in 72.9% of the women and the mean age of the menopause was 48.4 ± 5.2 years. The PMSQ with 36 items demonstrated that this questionnaire is an adequate instrument to evaluate sexual dysfunction in menopausal women. The correlation between the FSFI, used as a gold standard, and the PMSQ was high (r = 0.79; *p* < 0.001). The PMSQ cutoff point was established as ≤ 55.6, assuming a sensitivity of 87.9% and specificity of 78.9% (*p* < 0.001). The PMSQ identified 54.7% of the women with sexual dysfunction and, when the FSFI was used, that proportion was 50.3%.

The current study has several strengths. Factor analysis assured that the PMSQ fit the theoretical concepts of Basson female sexual response cycle.^28^ ^33^ ^34^ In addition, the factor loading of the individual items met the expected standard, supporting the factorial validity of this instrument. The results met the statistical requirements of the factorial structure and the internal consistency of the total instrument and its domains were high. Another aspect to be considered is that the criterion validity was verified using the gold standard FSFI questionnaire.^35^ The PMSQ also has the ability of measuring both peripheral (lubricating) and central sexual response (arousal, desire), important domains for assessing sexual response, such as sexual intimacy and self-image.

Among the potential limitations of the present study, the number of participants of five per item was close to the average that is recommended.^28^ Another drawback was the low socioeconomic level of the population included in the study, and the low level of education. In addition, the researcher needed to conduct the interviews face to face. Therefore, for external validation, the authors are aware of the need to examine the applicability of the instrument to other populations with different socioeconomic levels and different levels of education.

Moderate correlation was found between the domain influence of menopause in the PMSQ and all FSFI domains, except for the pain domain, which showed weak correlations with almost all other domains. The low correlation between the FSFI pain domain and the other PMSQ domains suggests that pain during sex may be only slightly related to the sexual response components. These results are in accordance with the theoretical framework adopted for the construct, in which the sexual response involves a coordinated sequence of several phases, including desire, arousal, orgasm, and sexual intimacy.^6^

A study conducted in Brazil with 540 women, at the ages between 45 and 60 years old with sexual dysfunction has shown association between lubrication condition and sexual dysfunction, but those who presented satisfaction in the relationship to their partners had lower sexual complaints.^36^ Sexual intimacy domain in the questionnaire assesses intimacy with the partner during sexual intercourse and many older women maintain sexual satisfaction because of the protective role of the psychosocial factors clearly associated with a happy relationship. In a postal survey conducted in Australia, the relationship factors had a more negative impact on desire than the age or menopause condition. This same study showed that physiological and psychological factors may be more significant for low genital arousal and low orgastic function.^37^ The moderate but significant correlations between sexual intimacy (r = 0.40) with desire, satisfaction, arousal and a weak correlation with menopause (r = 0.20), orgasm (r = 0.30) and self-image (r = 0.30) in the current study support the knowledge that sexual intimacy is an important factor and should weigh among the domains of any instrument designed to evaluate the female sexual function.

A general decline in postmenopausal self-esteem and well-being may also contribute to the loss of sexual intimacy with the partner.^38^ The physical and psychological symptoms of menopause and the simultaneous decline in sexual function may result in an inferiority sensation and negative body image in postmenopausal women, thus reducing their quality of life.^39^ The self-image domain, being directly related to self-esteem, showed a better correlation with sexual intimacy in the present study, but no significant correlation with sexual quality of life and menopause domains. In the group of women classified with sexual dysfunction, the PMSQ showed the lowest mean score in the menopause domain, indicating that the menopause condition itself has a negative impact on the sexual response cycle, sexual quality of life, arousal and desire. In the analysis of the FSFI scores, except for the menopause domain, the domains that obtained low scores the most in this population were the desire and arousal domains.^12^

## Conclusion

The psychometric validity of the PMSQ, including construct and criterion validity, responded satisfactorily to the tests performed. The current instrument showed adequate factor loadings, good internal consistency, and high coefficient correlation with the gold standard instrument. Therefore, the evaluation of sexual dysfunctions during menopause has a valid and reliable instrument that includes specific domain. The PMSQ can be used to examine sexual function in postmenopausal women, but further studies in other populations with different social levels and lifestyles are needed.
